# Harnessing iPSC technology for population-level human disease/traits modeling -where are we?

**DOI:** 10.3389/fcell.2026.1927966

**Published:** 2026-08-12

**Authors:** P. K. Suresh

**Affiliations:** School of Bio Sciences & Technology, VIT, Vellore, India

**Keywords:** diversity-in-a-dish, humanity-in-a-dish, induced pluripotent stem cells (iPSCs), population and cell-based modeling, quantitative expression loci (QTL), single cell RNA sequencing (scRNA), village-in-a-dish

## Abstract

This mini review highlights the major developments in iPSC research and the importance of dissecting the contribution of epigenetic factors in resetting the epigenome and their correlation to the stage-wise transitions to the dedifferentiated state. Since the need of the hour is to identify multiple genetic variants at the population level that are the major contributors to the heterogeneity in the inter-individual response (combination of environmental and/or genetic influences), it is necessary to adopt methodologies (in human iPSC cell lines) including DNA sequencing methods, SNP genotyping arrays, and other variants identification methods to identify, quantify these micro-level changes and correlate them with the disease phenotype. Further, studies (including scRNA-based approaches) need to be performed to compare variants at the transcriptomic level to trace it back to the specific individual/human cohort, thereby unraveling the context-dependent masked effects at the population level and fill in the gaps between the clinical aspects and e-localization signals. Further, village-in-a-dish (co-culture of heterogeneous cell types differentiated from the different iPSC cell lines isolated from human donors) and humanity-in-a dish model strategies will benefit from ongoing methods to generate an atlas of genetic variants that can explain genetic diversity globally. Parallel efforts to improve the scalability, automation and throughput in the development of mature human cells as well as refine strategies to better correlate and perform QTL mapping studies will complement the existing body of clinical and molecular data and unravel the relative role played by environmental factors to the overall disease risk, phenotype and drug response.

## 1 Introduction

This review highlights the significance and remarkable potential of iPSCs (isolated from different human donors) that provides a fairly unlimited source of material that can be used to model and better understand inter-individual differences (not entirely possible by analyzing genetic variant data currently available in the databases as well as correlated clinical findings) in terms of disease susceptibility, disease phenotype and drug response. This is primarily the case due to diseases being generally polygenic with multiple genes controlled by genetic variants (empirically associated with human disease) and expression Quantitative Trait Loci (eQTL) variant regulating gene expression (in cis or in trans) that can be further modulated by several environmental factors. Due to the multi-factorial nature of complex human diseases and traits, it is necessary to capture the entire spectrum of regulatory changes (in coding and non-coding regions) that contribute to the diversity in genetic factors that, in major part, are known to contribute to the observed heterogeneity in human populations. In this regard, this review highlights the importance of humanity-in-a-dish; diversity-in-a-dish and village-in-a dish models using iPSCs (from defined human cohorts with an adequate sample size) that offer an unprecedented opportunity to co-culture heterogeneous cell types simultaneously and correlate RNA sequencing data with certain genetic variants (like SNPs) to map eQTL. Further, it is necessary to perform molecular studies at the single cell level to disentangle the various mechanisms that contribute to the disease phenotype as a consequence of multiple environmental influences, as well as pinpoint the exact genetic region that may be causative (to overcome linkage disequilibrium-related issues). Despite major advancements, this review identifies challenges remain in terms of scalability, generalizability, automation and the production of reproducibly mature, differentiated cells as well as in terms of mass sequencing efforts to document genetic diversity in several human populations globally, apart from precisely deciphering subtle changes in gene expression and the resultant multiple context-dependent effects influencing the final integrated biological response.

## 2 Key historical developments in iPSC research & reprogramming efficiency

Sir John Gurdon developed tadpoles by transplanting the nucleus from an adult differentiated epithelial cell (from *Xenopus laevis*) into an enucleated frog egg cell. This groundbreaking finding showed that the nucleus has the genetic information necessary for reprogramming the enucleated cell. The next major development involved the cloning of Dolly the sheep by Ian Wilmut and other scientists. Subsequently, the work of Tada et al. (2001) resulted in the production of pluripotent heterokaryons (fusion of ESCs with the somatic thymocyte -4N -tetraploid), that were reprogrammed by the factors present in the ESCs. The revolutionary work for iPSC development was performed by Takahashi & Yamanaka, wherein they demonstrated the induction of pluripotency (in human fibroblasts) using defined, retrovirally transduced, shortlisted transcription factors (Oct4, Sox-2, Klf-4 and c-Myc), while Thomson’s group achieved the same result by employing Oct-4, Sox-2, Nanog, and Lin-28 (Cerneckis et al., 2024). However, one of the major challenges involves reprogramming efficiency and the possible risk of insertional mutagenesis (inactivating an essential gene) and/or the possible integration of the gene(s) encoding for the Transcription factors and their residual expression (during the reprogramming factor independent phase of iPSC generation). In order to improve safety, non-integrated methods have evolved and they include Adenovirus, Sendai virus, coding and noncoding RNA-based delivery as well as CRISPR-based methodologies. A recent paper, using 150 iPSC cell lines, demonstrated Sendia virus-based reprogramming as the method of choice in terms of reprogramming efficiency (across different methods), while mRNA-based transfection yielded the best result when fibroblasts were used as the cell type. This finding also underscored the importance of immunological and donor-specific factors influencing the final outcome, apart from epigenetic factors responsible for silencing somatic genes and the reactivation of those associated with pluripotency (Kuebler et al., 2026). An improved understanding of these factors would enhance our ability to optimize methods to enhance the reprogramming process for the possible development of patient-specific stem cells (for regenerative therapies, model development and drug testing).

## 3 Epigenetics of pluripotency, epigenetic memory and safety

Since resetting of the epigenome is a critical determinant for the re-acquisition of pluripotency, it is necessary to obtain an in-depth understanding of the various epigenetic mechanisms that govern this process, wherein the cell identify and cell fate is altered and the network of genes linked to the embryonic as well as the pluripotent states are re-expressed. A paper outlines the steps involved in the conversion of the differentiated cell to the pluripotent state including the salient aspects of the intermediate cellular and molecular states. thereby providing information that can also facilitate trans-differentiation processes. In addition, knowledge of partial-reprogramming has implications in the reversal of cellular aging, wherein resetting occurs without a loss of cellular identity (Papp and Plath, 2011). An alternative method, to achieve pluripotency, involves chemical molecules-based reprogramming has been described. In this study, immunofluorescence studies were adopted to track the different stages of progressive cell dedifferentiation (epithelial-like -stage I); intermediate plastic-like, XEN-like state, primary cells (human chemically induced pluripotent stem cells -hCiPS cells). The different stages were correlated with markers characteristic of the respective states (Stage I-LIN28A; Stage II -LIN28A, SALL4; Stage III -SALL4, OCT4; Stage IV -OCT4, SOX2, NANOG). This work was made possible based on the availability of a discrete combination of chemical molecules (especially JNK inhibitor that in essential for reprogramming) that can facilitate the conversion of the differentiated cell into cells of the different stages based on the sequential activation of pluripotency genes and expression of dedifferentiation-related genes (Guan et al., 2022). A transient, naïve treatment protocol has been developed that can reset the epigenome in the cells in terms of memory-related marks and corrects the aberrations to create a temporary pluripotent stem cell state that matches the epigenome of hESCs (Buckberry et al., 2023). However, still problems related to heterogeneity, persistence of Sendai virus and continued expression of transcription factors as well as errors in terms of the timing of DNA replication need to be addressed. Progress continues to be made in term of reducing the risk of cancer formation and these strategies include improving iPSC cell purity; adoption of integration-free strategies for introducing the genes coding for key transcription factors; optimize reprograming cocktail of molecules (genes encoding for proteins, proteins, mRNA and noncoding RNA) as well as small molecules) as well as suicide-gene technology to eliminate the potentially tumorigenic cells (Zhong et al., 2022). Further, immunogenicity concerns, with allogeneic hiPSC therapy, can be addressed by knocking out genes coding for certain MHC molecules as well as increase the expression of immunomodulatory molecules (Asadi-Sarabi et al., 2025). The section below outlines the rationale for iPSC-based studies to correlate genetic variants and eQTLs and pQTL with the disease risk phenotype for complex human disease traits.

## 4 Cell line-based studies -immortal cell lines to iPSCs to village-in-a dish model

The limitations imposed by species differences with animal model systems and ethical concerns has necessitated the need to perform mechanism-based studies using immortalized cell lines (all primary cells cannot be immortalized). Data generated from such models has limited utility, since they frequently do not reflect the entire pathologic mechanistic processes in humans, as well as being unable to reproduce the entire spectrum of genetic and epigenetic changes observed at the population level.

Some of these limitations in terms of genetic, epigenetic and microenvironment-related heterogeneity are being overcome with the development of higher order *in vitro* spheroid and organoid models as well as organ-on-a-chip and whole-body model systems. In parallel, the availability of vast amounts of human disease clinical data in databases permits correlations to be done, based on GWAS (Genome Wide Association Study -population level links between DNA variants and disease risk/drug response based on statistical correlations) and Quantitative Trait Loci (QTL)-based approaches (how these genetic variants genetically control gene expression at the RNA and/or protein levels) with complex human disease phenotypes (Warren and Cowan, 2018). In the case of GWAS studies, whole genome sequencing and/or SNP-based genotyping arrays and other assays for non-SNP-based variants) are employed, while RNA sequencing and mass spectrometry/antibody-based assays are adopted for expression QTL (eQTL) and protein QTL (pQTL) studies respectively.

It was therefore necessary to develop experimental designs, wherein both the genetics as well as the mechanisms underlying human biology/pathology could be studied. In this regard, a large-scale study was performed using a combination of whole genome data from the 1000 genomes project involving an African and European population as well as RNA sequencing, (data obtained from matched lymphoblastoid cell lines). eQTL mapping (cis and trans) for common and rare regulatory variants (outlier analysis) was done and links were established between GWAS-based genetic variants, alterations at the RNA level with certain complex human disease traits. This analysis also discovered that the majority of the expression variants were novel, (when correlated with HAPMAP3 data), enriched in splice sites and in nonsense variants and this reference dataset has provided opportunities for future comparative studies wherein genetic variants will be correlated with transcriptomic and clinical data for polygenic complex human diseases with a multifactorial etiology (Montgomery et al., 2011). Methods standardized herein can be extended to iPSC cell lines which provide a better option (instead of lymphoblastoid cell lines) for both mechanistic studies and drug development. These relatively undifferentiated cell lines can model and correlate small and large effect-related traits in the different human developmental stages as well as transient expression states in rare and complex genetic disorders. In this regard, it is pertinent to point out that existing iPSC cell lines will obviate the costs and times involved in identifying a cohort of humans exhibiting a particular disease phenotype as well as standardizing and optimizing conditions for proliferation reprogramming and quality control. of human iPSC cell lines. However, certain genetic traits eQTL and pQTL are expressed only in certain experimental conditions (“context-dependent effects” -discussed below), thereby warranting the need to analyze several cohorts of iPSC cell lines (produced after a human cohort has been identified and recruited), despite the process being costly and lengthy. Further, inherent cell-type and context-specific variability being masked as a consequence of bulk RNA sequencing and batch effects, has resulted in an imperative need to develop single cell level studies.

Inter-individual differences have posed significant challenges for identifying a suitable cohort of humans and standardization of protocols for inter-laboratory validation of data using cryopreserved and freshly prepared cells. In order to identify the major sources for this variation, a study was performed wherein 1–2 cell lines/donor were short-listed based on a combination of cellular and molecular assays. This selection was also based on pluripotency markers as well as markers that are representative of the cells of the three germ layers. Finally, selection of the 1–2 cell lines was made based on results obtained from a combination of assays (RNA-seq, DNA methylation arrays, quantitative proteomics and cell morphological imaging). It was found that a significant proportion of the variability in the cellular and molecular phenotype could be correlated with genetic factors (regulatory sequences found distally controlling eQTL-based expression profiles), with Copy Number Alterations (CNAs) also having a role. Further, a subset of eQTLs (found in pluripotent “stem cell-like cells”) can be used to flag certain loci that are linked to certain development states and human disorders like cancer (Kilpinen et al., 2017). In a more recent study involving 1367 human pluripotent cell lines and mapping at the population level with increased statistical power, the methodologies developed earlier (Montgomery et al., 2011) were employed to statistically correlate cell-type specific transcriptomic data with common and rare eQTL-based information, thereby enabling a link to be established between these variants and complex human traits (Bonder et al., 2021).

## 5 Context-dependent effects

Apart from the correlated genetic factors with clinical phenotypes, it is necessary to unravel the relative contribution of environmental influences that regulate gene expression in iPSC cell lines, that better capture the inter-individual differences based on cell type-specific regulatory mechanisms (cis and trans-QTLs mainly in the noncoding regions). In order to evaluate this type of variability, scientists differentiated macrophages from iPSCs harvested from 209 healthy volunteers. These differentiated cells were exposed to twelve different exposure conditions that mimicked the “real-life” scenario in terms of infection and inflammation and RNA-based changes in gene expression were determined (from 209 unique iPSC cell lines -two different time points −24 data points). It was observed that among the 1955 disease -eQTL co-localization events (from 83 studies of sufficient power), 51% of regulatory effects observed were not detectable in GTEx TISSUE (Gene Tissue Expression Database), thereby substantiating the need for condition-specific gene expression measurements (reQTLs) with concomitant co-localization of disease-associated gene loci (based on population level data). Due to the fact that reQTLs were, in major part, localized to genes that did not vary considerably in expression level in the basal and stimulated states, it can be inferred that they may induce small changes in gene expression and yet contribute to meaningful variations in the final biological response. Nevertheless, reQTLs have accounted for 21.7% genes linked to diseases at GWAS loci, that were not detected based on gene expression studies conducted at basal conditions. However, for detecting and pinpointing trait-specific genes for complex diseases, micro-level eQTL changes (resolve linkage disequilibrium-related issues and to identify the entire spectrum of eQTL-related changes) need to be detected in a relatively much larger set of samples with sufficient statistical power. Hence, the limitations of this study need to be recognized in terms of sample size constraints, inability to fully capture the entire spectrum of regulatory changes, interplay between epigenetics and transcription factors, dynamics of chromatin assembly and disassembly as well as splice-site related changes (Panousis et al., 2025). In a companion paper, it was found that the co-localization gap between GWAS data and eQTL variants in terms of the inability to explain Immune-Mediated Disease (IMD)-related risk could be filled, at least in part, by determining differences in splice site usage (mainly in introns and especially those that were not used that often) and correlating them with GWAS variants. Specifically, macrophages, differentiated from hiPSCs, were exposed to environmental stimuli and alterations in alternative splicing were detected. There were significant differences between the control and exposed groups (over 5734 genes across all the experimental conditions -high effect size). Correlation was done for over 700 genes (significant with a high effect size and hence considered to be response splicing (sQTL) and IMD related disease loci for 22 IMDs. This approach will aid in the deciphering the consequences of such splice site variants at the transcriptomic level, apart from assessing therapeutic strategies targeting this machinery selectively. Future studies warrant a quantification of isoform-specific transcript variants as well as functional studies for an improved comprehension of linkage disequilibrium in disease-related loci as well as the role of such variants in complex disease processes (El Garwany et al., 2025). There is also a discovery bias related to all the aforesaid discussed strategies, due to differences between GWAS variants and eQTL variants influenced by natural selection processes. This bias may be attributable, to the fact that GWAS variants are enriched in more vital genes in which mutations are not tolerated and those that are responsible for pleiotropic functions. Further, their regulatory processes are complex and are enriched predominantly in regions regulating transcription factors. eQTL variants, on the other hand, do not exhibit the aforesaid features and occur mainly in promoter regions. Hence, apart from GWAS-based variant analysis along with relevant eQTL-based studies and splice site analysis (all can be subject to discovery bias), other assays, wherein regulatory portions of the genome can be predicted by studies based on DNA sequence information, need to be incorporated. Such a combination of techniques, including functional assays like CRISPR-based gene editing/enhancer silencing approaches, can provide opportunities for better correlation between the different types of variants and the disease phenotype (Mostafavi et al., 2023).

Since a tissue has multiple, differentiated, heterogeneous cell types, a better model system is needed that can more faithfully recapitulate the heterogeneity (genetic factors and an interplay with environmental factors) contributing to the complex disease phenotypes/traits. In this regard, one of the significant revolutionary strategies involves the development of village-in—a dish model, wherein a mixture of different genetically different induced pluripotent stem cell lines is simultaneously co-cultured in the same experimental *in vitro* reaction vessel. The validation of this type of model stems from the reproducibility (scRNA sequencing to better determine the heterogeneity in RNA expression) demonstrated with respect to the gene expression data, including correlated eQTL-based context-dependent effect data obtained from single cell lines (monoculture) as well as from village-in-a-dish model system. Also, results were reproducible in different laboratories and barring some variation in the expression of some stem cell markers (MYC, NANOG, SOX2 and POU5F15), correlations were obtained in data generated from cryopreserved as well as from freshly prepared iPSC cell lines. Hence, village-in-a-dish model represents a major advancement in the robust and reproducible modelling human disease-relevant, genetic and epigenetic risk and mechanistically-linked factors providing opportunities to uncover dynamic, context-dependent effects. However, inherent limitations pertaining to differences in growth rate of the respective human iPSC cell lines, possible experimental artifacts due to possible inaccuracies in terms of *in vitro* differentiation as well as these models being incomplete surrogates of the entire organ/entire human body need to be addressed (Neavin et al., 2023). As an extension of this concept, neural progenitors were developed (Stem-cell-derived NGN2-accelerated Progenitors (SNaPs) developed from human iPSCs from different donors) and pooled to develop a “village-in-a-dish model” for assessing the effects of gene variants (generated based on CRISPR-knockout technology) on the response of cells to a challenge zika virus infection, thereby modelling (*in vitro*) gene-environment interactions that can be extended to more assess specific genetic variants and the risk of neurological diseases (Seah and Brennand, 2023). Due to the heterogeneous nature of iPSC cell lines, for the incorporation of “diversity-in-a-dish” model, a decision tree needs to be incorporated in the study design based on certain criteria (reason for the experiment to evaluate population level variation; background information providing evidence that diversity influences the disease process; model good enough to differentiate the “signal” from the “noise” and has sufficient statistical power (Weidema et al., 2026). This approach can possibly be applied to experimental designs involving developing a disease-in-a-dish model using differentiation protocols and strict quality control procedures, as well as functional and high content imaging assays, ultimately culminating in trial-in-a-dish experiments (Warren and Cowan, 2018). Such imaging-based Cell painting screening assays that can be automated and multiplexed, have been employed to simultaneously analyze drug effect in pluripotent hiPSC cell lines (isolated from the HipSci repository that consists of >700 cell lines from various donors) and document an important finding in terms of inter-individual variability in the phenotypic responses. Further, the cell painting data were linked with mass spectrometry-based analysis for a better mechanistic understanding of the factors that may have contributed to the heterogeneity in drug response. This approach provided a platform for comparing the safety of drugs in cell lines isolated from human populations with diverse genetic features (Platani et al., 2025). Such toxicity screens can include screening drugs with hepatotoxic and cardiotoxic potential involving multiple high throughput testing methods in multiple organ model systems that can better recapitulate the multi-factorial pathological process (Friese et al., 2019).

Among the different determinants for accurate modelling of the disease phenotype, the maturation status of the fully differentiated iPSCs should recapitulate the *in vivo* state and is of paramount importance also in terms of modelling dynamic eQTLs that may regulate only the intermediate stages (Strober et al., 2019), and can contribute to inter-individual differences in terms of reproducibility and generalizability/validity of our findings. Further, automation of the assays will expedite the experiments that will aid in the mechanistic correlation between data generated at the population with single cell-based molecular findings (that can be extended to include other types of QTLs) (Arthur et al., 2025). In this regard, global transcriptome level understanding of the similarities and differences (genetic and non-genetic factors) can be obtained based on a systematic comparison of single cell data from primary cells, ESCs and hiPSC lines using statistical approaches like Principal Component Analysis (PCA) (Zhang et al., 2024) by adopting R and Python-based workflows. Also, such comparative approaches can be complemented by the utilization of certain R packages (for, e.g., pathlinkR) that aid in the transcriptomic analysis of differentially expressed genes using pathway enrichment strategies and network-based approaches, that can possibly aid in a more accurate identification of the molecular mechanisms underpinning the various human pathologies (Blimkie et al., 2024).

Existing challenges include comprehension of the cellular changes due to micro-level changes in gene expression (not discernible clinically), the myriad different environmental factors that can singly and/or in combination modulate the underlying genotype as well as generate data that can provide an atlas of genetic variants contributing to global diversity. Ongoing strategies to address these lacunae include increasing the throughput and improving quality control practices for iPSC derivation, propagation, and differentiation/maturation that can also aid in the creation of genetically diverse, globally relevant iPSC biobanks. These biobanks will help in cataloguing effect size at the cellular and molecular level and for tracing it back to clinical data (for, e.g., fMRI for correlated neuronal function and assessment of the effect at the cellular and clinical level with a spectrum of variants (SNPs and other variants) generated from correlated, defined human cohorts) (van Zanten et al., 2026).
